# Waldenström Macroglobulinemia-Associated Peripheral Neuropathy in the Brachial Plexus With Bing-Neel Syndrome Diagnosed Through Repeated Cerebrospinal Tests

**DOI:** 10.7759/cureus.52400

**Published:** 2024-01-16

**Authors:** Misaki Hatasa, Naoto Imoto, Shota Komori, Yasunobu Nosaki, Shingo Kurahashi

**Affiliations:** 1 Department of Postgraduate Clinical Training Center, Toyohashi Municipal Hospital, Toyohashi, JPN; 2 Department of Hematology and Oncology, Toyohashi Municipal Hospital, Toyohashi, JPN; 3 Department of Neurology, Toyohashi Municipal Hospital, Toyohashi, JPN

**Keywords:** central nervous system disease, waldenström macroglobulinemia-associated peripheral neuropathy, tirabrutinib, bruton’s tyrosine kinase inhibitor, bing-neel syndrome

## Abstract

In Waldenström macroglobulinemia (WM), confirming the presence of Bing-Neel syndrome (BNS) is important because drugs that penetrate the central nervous system (CNS) must be selected. We report the case of a 75-year-old man for whom tirabrutinib, a second-generation Bruton’s tyrosine kinase inhibitor (BTKi), was useful in treating WM-associated peripheral neuropathy (PN) with BNS. Numbness and muscle weakness in the fingers occurred three years after the initial treatment of WM. WM-associated PN due to demyelinating disease was diagnosed based on the results of a nerve conduction study and magnetic resonance imaging showing bilateral symmetric swelling of the brachial plexus. The cerebrospinal fluid (CSF) cytology results were initially negative; however, the CSF test was repeated because of extremely high protein levels (984 mg/dL) and slightly elevated leukocyte counts (14/µL). The second test revealed abnormal lymphoplasmacytic cells (189/µL), indicating BNS. Rituximab and high-dose methotrexate-containing chemotherapy were administered. Despite the subsequent negative CSF cytology results, his neurological symptoms persisted but subsided soon after the initiation of tirabrutinib. The therapeutic effects of tirabrutinib persisted for 25 months. This case suggested that a careful search for concurrent BNS is important when lesions are close to the CNS or when atypical CSF findings are obtained in patients with WM-associated PN, especially when BTKi options are available.

## Introduction

Peripheral neuropathy (PN) occurs in approximately 20% of patients with Waldenström macroglobulinemia (WM) at diagnosis and in up to 50% of patients at some point during the progression of the disease [1]. In contrast, Bing-Neel syndrome (BNS) is a rare form of WM with lymphoplasmacytic infiltration of the central nervous system (CNS), accounting for approximately 1% of all WM cases [2,3].

Despite several treatment options available for WM-associated PN, there is no trial data specifically assessing the efficacy of these options [1]. Although ibrutinib has been reported to be effective against WM-associated PN [4,5], the efficacy of second-generation Bruton’s tyrosine kinase inhibitors (BTKi) in WM-associated PN remains unclear. Determining the occurrence of BNS is important for treatment selection because BNS is treated with drugs that need to penetrate the CNS. Case reports have suggested that tirabrutinib is effective against BNS [6-10].

Herein, we report a patient diagnosed with WM-associated PN who also presented with BNS based on several modalities, including repeated cerebrospinal fluid (CSF) tests. Following rituximab, high-dose methotrexate, procarbazine, and vincristine (R-MPV) therapy, the patient's symptoms were resolved with tirabrutinib treatment. We also reviewed five previously reported cases of BNS treated with tirabrutinib [6-10].

## Case presentation

A 75-year-old man developed numbness and muscle weakness in the fingertips of both hands during follow-up after treatment for WM. Four years ago, he was diagnosed with WM owing to high serum levels of immunoglobulin (Ig) M and IgM-κ type M-protein and increased numbers of MYD88 L265P mutation-positive plasma cell-like lymphocytes in the bone marrow. Therapy with dexamethasone, rituximab, and cyclophosphamide was initiated due to progressed pleural effusion, but no treatment response was observed. Bortezomib was subsequently administered, and a complete response was achieved.

His neurological symptoms gradually worsened over a period of three months, at which point we started the assessment. Physical examination revealed intrinsic muscle atrophy in both hands and decreased deep tendon reflexes in the upper extremities. The hand grip was also reduced in both hands (right, 13 kg; left, 7 kg). A nerve conduction study revealed proximal conduction velocity delay and conduction block in the median and ulnar nerves, suggesting extensive demyelinating disease. CSF tests showed increased levels of CSF protein (984 mg/dL), slightly increased leukocyte count (14/µL), unclassifiable cells (1/µL), and increased immunoglobulin levels (IgG: 70.3 mg/dL, IgM: 58.9 mg/dL); however, cytology was negative. The presence of anti-myelin-associated glycoprotein (anti-MAG) antibodies was not tested because this examination is not covered by health insurance in Japan.

**Figure 1:**
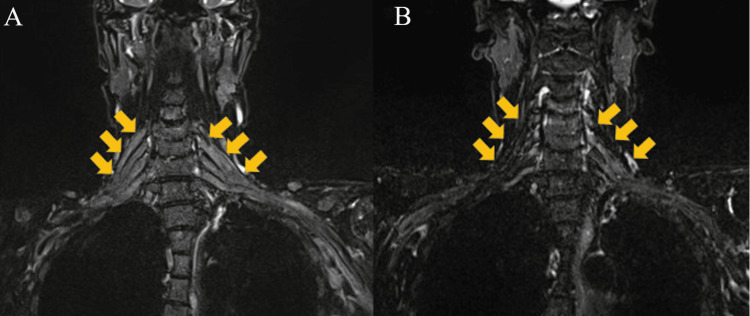
T2-weighted coronal magnetic resonance imaging (MRI) of the cervical area after diagnosis of Waldenström macroglobulinemia (MW)-associated peripheral neuropathy (PN) Yellow arrows indicate the brachial plexus. (A) Before treatment for PN and (B) 15 months after treatment with tirabrutinib.

**Figure 2:**
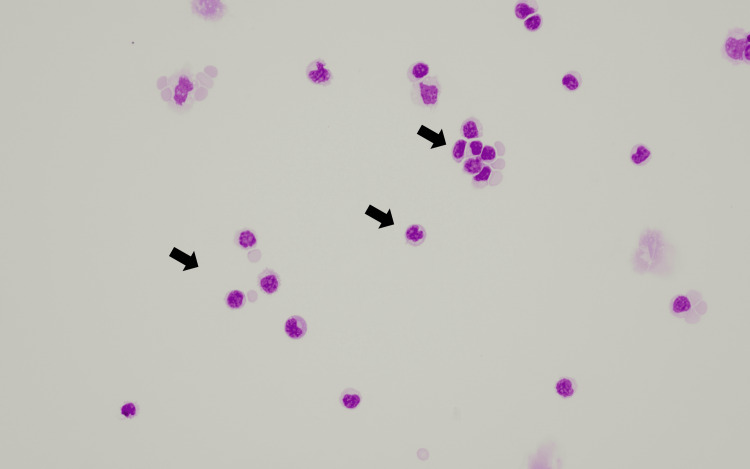
Cerebrospinal fluid (CSF) image at the diagnosis of Bing-Neel syndrome (BNS) (Giemsa staining, ×400)

In Figure 1, magnetic resonance imaging (MRI) revealed diffuse bilateral symmetric swelling of the nerve root to the brachial plexus at the C4-Th1 level (Figure 1A) with normal brain, cervical, and thoracic spines. Although these findings were consistent with inflammatory demyelinating disease, we considered that the slightly elevated numbers of leukocytes and extremely high levels of the CSF protein were not indicative of immune-mediated neuropathy. A repeat CSF test three weeks after the first test revealed the presence of the IgM-κ type M protein and an increase in the number of abnormal lymphoplasmacytic cells (189/µL) (Figure 2).

However, the red blood cell counts and sugar levels were normal. In addition, the levels of serum IgM (885 mg/dL, reference range: 33-183 mg/dL) and C-reactive protein (2.81 mg/dL, reference range: 0.0-0.14 mg/dL) were also increased. Table 1 shows the most relevant test results for each phase after the diagnosis of WM-associated PN.

**Table 1:** The most relevant tests for each phase after the diagnosis of Waldenström macroglobulinemia (WM)-associated peripheral neuropathy (PN) *confirmed negative one month before CRP: C-reactive protein; IFE: immunofixation electrophoresis; IgM: immunoglobulin M; LDH: lactate dehydrogenase; N/A: not available; R-MPV: rituximab-methotrexate-procarbazine-vincristine; BNS: Bing-Neel syndrome; PN: peripheral neuropathy; CSF: cerebrospinal fluid

Variable	At diagnosis of peripheral neuropathy	At diagnosis of Bing-Neel syndrome	After two courses of R-MPV	At the completion of R-MPV	24 months after administration of tirabrutinib	Reference range
Complete blood cell count						
White blood cell (/μL )	5390	5470	10380	6520	4770	3300–8600
Neutrophil (%)	56	64.7	78	66.6	52.2	38.5–80.5
Lymphocyte (%)	25.4	19.2	11	16.6	36.7	16.5–49.5
Monocyte (%)	16.9	14.8	9	14.1	9	2.0–10.0
Hemoglobin (g/dL)	11.8	10.9	10.6	12.3	13	13.7–16.8
Platelet count (×10^4^/μL)	29	26.6	22.7	15.8	14.2	15.8–34.8
Blood chemistry						
LDH (U/L)	130	123	130	158	149	124–222
CRP (mg/dL)	2.58	2.81	0.04	0.51	0.1	0–0.14
IgM (mg/dL)	832	885	306	168	120	33–183
Cerebrospinal fluid						
Red blood cell (×10^4^/μL)	0.01	0.01	0	0.02	0.01	-
Leukocytes (/μL)	14	184	15	6	5	-
Mononuclear cells (/μL)	14	183	15	5	5	-
Unclassifiable cells (/μL)	1	6	1	0	1	-
Protein (mg/dL)	984	572	312	98	54	-
Glucose (mg/dL)	38	88	63	45	50	-
IgM (mg/dL)	58.9	N/A	12.8	1	0.2	-
M protein (IFE)	N/A	IgM-κ M protein	N/A	N/A	N/A*	-
Cytology	negative	Plasma-cell-like lymphocytes positive	Negative	Negative	Negative	-

Neither a CSF MYD88 mutation test nor flow cytometry (FCM) were performed. The patient was diagnosed with WM-associated PN accompanied by BNS.

**Figure 3:**
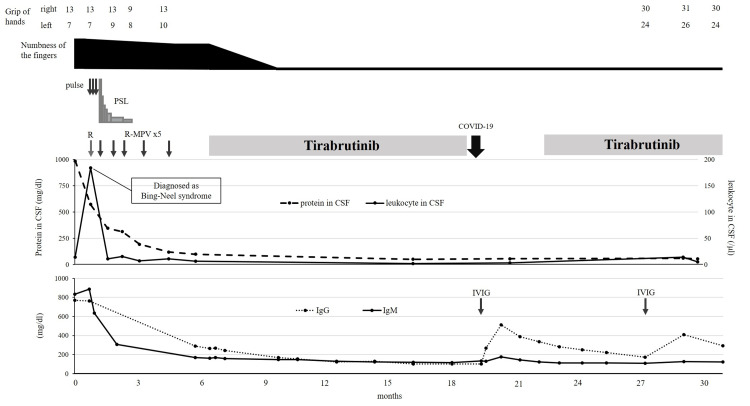
Clinical course of the patient after the diagnosis of Bing-Neel syndrome (BNS) CSF: cerebrospinal fluid; Ig: immunoglobulin; IVIG: intravenous immunoglobulin; PSL: prednisolone; R: rituximab; R-MPV: rituximab-methotrexate-procarbazine-vincristine; WBC: white blood cell

Four months after symptom onset, the patient was administered steroid pulse therapy (methylprednisolone 1000 mg/d for three days) and rituximab (375 mg/m2) to treat BNS complicated by inflammatory demyelinating disease, followed by R-MPV (day 1, rituximab, 375 mg/m2; day 2, methotrexate, 3.5 g/m2; and vincristine, 1.4 mg/m2; days 2-8; and procarbazine, 100 mg/m2 per day, administered during odd cycles). After two courses, the neurological symptoms of the patient were slightly improved, although the MRI revealed no improvement in brachial plexus swelling. However, a repeat CSF test showed a decrease in the levels of CSF protein and cell counts (protein, 312 mg/dL; leukocytes, 15/µL), suggesting a treatment response, and thus R-MPV therapy was continued. Evaluation of the treatment response after completion of the fifth course showed negative CSF cytology results and improved biochemical data (protein, 98 mg/dL; leukocytes, 6/µL; and serum IgM levels, 168 mg/dL). As the peripheral nerve symptoms persisted, tirabrutinib therapy (480 mg/d) was initiated. The patient showed improvement in his symptoms one week after starting tirabrutinib treatment. However, he developed grade 1 eczema two weeks after the initiation of tirabrutinib therapy, which was resolved after treatment with a steroid ointment. Before starting tirabrutinib, the patient had difficulty with movements requiring dexterity and muscle strength, such as buttoning, using chopsticks, and wringing towels. However, the patient was able to perform these activities normally for several months after the administration of tirabrutinib. Tirabrutinib was administered for 12 months but was discontinued when the patient developed a COVID-19 infection. The levels of serum IgG in the patient were gradually decreased to <100 mg/dL after treatment initiation. To prevent the worsening of the COVID-19 infection, an immunoglobulin preparation was administered. Tirabrutinib treatment was subsequently resumed. Re-evaluation before the resumption of tirabrutinib treatment (15 months after the initial treatment with tirabrutinib) confirmed a reduction in the pretreatment swelling of the nerve root and brachial plexus (Figure 1B). The patient was followed up for 25 months from the start of tirabrutinib therapy, exhibiting improved hand grip and no deterioration of neurological symptoms compared with before treatment (right: 13 kg → 30 kg, left: 10 kg → 24 kg). The clinical course of the patient is shown in Figure 3.

## Discussion

In the present case, we diagnosed coexisting WM-associated PN and BNS, despite the initial negative results for BNS. The patient had motor and sensory dysfunction of the upper extremities with symmetric symptoms mainly localized to the fingertips, which are typical PN symptoms. Diffuse bilateral symmetric swelling of the nerve root to the brachial plexus, with a normal brain and cervical and thoracic spine on MRI, was also consistent with PN [1]. Thus, we assumed that these symptoms were due to WM-associated PN, with the patient being asymptomatic for BNS. To the best of our knowledge, the present case may represent the first report of repeated CSF tests revealing WM-associated PN accompanied by BNS.

Various factors might cause WM-associated PN, including anti-MAG antibodies, rare autoantibodies, neurotoxicity due to direct tumor infiltration, amyloidosis caused by WM, cryoglobulinemia, vasculitis, and chemotherapy [11]. Unfortunately, we were restricted by regulations in the measurement of anti-MAG and other antibodies related to PN due to the insurance system in Japan. We assumed that the immune-mediated mechanism was mainly attributed to diffuse, symmetric, and demyelinating patterns [1]. However, nerve root swelling and BNS suggested the possibility of direct infiltration of tumor cells into the nerves, which subsequently extended into the CSF.

The incidence of BNS could be underestimated because WM-associated PN is common, whereas the clinical symptoms of BNS are variable [2,3]. To prevent underestimation, it is important to consider atypical findings of WM-associated PN. Asymmetrical distribution, predominant motor dysfunction, balance disorders, gait abnormalities, cognitive impairment, and cranial nerve deficits are signs of suspected BNS [12]. As for the laboratory findings, inflammatory demyelinating disease is characterized by normal leukocyte counts of <10 mm2 [13], which in our case were slightly higher at 14/µL. In addition, inflammatory demyelinating disease is generally characterized by elevated levels of the CSF protein; however, a level of approximately 1000 mg/dL is not typical [14]. The difference in the value between the first and second CSF tests may be attributed to disease progression or a false negative in the first CSF. False-negative CSF cytology results are common, especially with small volumes (<10.5 mL) or a limited number of tests (<2) [15].

Once a diagnosis of BNS is suspected, it is recommended to have brain and entire spine MRI scans with gadolinium (Gd) administration. The MRI protocol should include fluid-attenuated inversion recovery and T1-weighted sequences before and after Gd injection. Lumbar puncture should be performed after MRI evaluation to avoid obstructive hydrocephalus and non-specific enhancement. The standard diagnosis of BNS is the demonstration of WM cells in the CSF, or less frequently, on brain tissue biopsy. FCM tests of the CSF, which are characterized by positive expression of CD19, CD20, CD22, CD79a, CD27, and CD52 and usually negative expression of CD5, CD10, and CD23, along with tests for immunoglobulin heavy chain gene rearrangement and MYD88 L265P gene mutation, are all important in supporting the diagnosis of BNS [12].

The diagnosis of BNS in our patient was limited to CSF cytology findings only, whereas FCM and genetic testing of CSF, which are required for diagnosing BNS [12], were not performed. However, considering the presence of lymphoplasmacytic cells in the CSF, the normal red blood cell count in the CSF, and the lack of WM cells in the peripheral blood at that time, a diagnosis of BNS would be appropriate [3]. Our case demonstrated that a careful evaluation of progression or coexistence with BNS is important when lesions are close to the CNS in PN or when the initial CSF findings are atypical in patients with WM-associated PN.

The goal of the treatment in BNS should be to reverse the patient’s clinical symptoms and prolong progression-free survival. However, it is unclear whether asymptomatic patients with BNS should receive treatment. The standard treatment for BNS has not been established, and several options, including high-dose methotrexate, high-dose cytarabine, bendamustine, cladribine, fludarabine, and ibrutinib, are present [12]. Some experts recommend reserving a high-dose regimen until relapse owing to its toxicity and setting ibrutinib in a frontline setting [12]. The treatment effect of BTKi for BNS has been previously reported with ibrutinib [2]. However, other studies reported the occurrence of some adverse events (AEs), such as atrial fibrillation and hemorrhagic events [4]. Tirabrutinib is a second-generation BTKi with a low affinity for kinases other than BTK that was designed to improve the efficacy and reduce the toxicity of BTKi [16]. Tirabrutinib was well tolerated in patients, with the most frequent AE being exanthema of grade 2 or lower, whereas no arrhythmia was observed [17]. Furthermore, preclinical data showed that among second-generation BTKi, tirabrutinib was transferred to the CNS more favorably than zanubrutinib [18], suggesting that tirabrutinib is a potent option for patients with central nervous lesions. This drug was first approved in Japan for the treatment of primary central system lymphoma [19] in 2020 worldwide, followed by its approval for use against WM [17].

In this case, preventing the relapse of BNS was possible due to the poor age-related prognosis and treatment for WM prior to BNS diagnosis [20]. A search for "Bing-Neel syndrome" and "tirabrutinib" in PubMed on December 3, 2023, identified five studies on tirabrutinib for treating BNS (Table 2) [6-10].

**Table 2:** Cases who had Bind-Neel syndrome (BNS) treated with tirabrutinib BD: bortezomib-dexamethasone; BNS: Bing-Neel syndrome; BR: bendamustine-rituximab; CR: complete response; CSF: cerebrospinal fluid; DRC: dexamethasone-rituximab-cyclophosphamide; EFS: event-free survival; F: female; HDAC: high-dose cytarabine; HD-MTX: high-dose methotrexate; Ig: immunoglobulin; IT: intrathecal; WM: Waldenström macroglobulinemia; M: male; MTX: methotrexate; N/A: not available; PD: progressive disease; PR: partial response; PSL: prednisolone; R-CHOP: rituximab-cyclophosphamide-adriamicine-vincristine-prednisolone; R-COP: rituximab-cyclophosphamide-vincristine-prednisolone; R-MPV: rituximab-methotrexate-procarbazine-vincristine; SD: stable disease; AEs: adverse events

Reference	Age at tirabrutinib administration (years)/sex	WM-related therapy before BNS diagnosis	Lesion at tirabrutinib administration	BNS therapy	Response of tirabrutinib	AEs of tirabrutinib	EFS months (from tirabrutinib administration)	Discontinuation of tirabrutinib
Yokoyama et al. [6]	46M	Cyclosporin, PSL, steroid pulse (treated as membranous nephropathy and inflammatory demyelinating disease	CSF, periventricular, full spine, bone marrow, kidney	Steroid pulse+ PSL → BR+ IT → [PD] → steroid pulse+ PSL → tirabrubinib	PR	(–)	15 months (no relapse)	(–)
Hagihara et al. [7]	62F	Rituximab monotherapy	CSF, Th2-6 thoracic spinal cord, left iliopsoas, and bilateral femurs	HDMTX → R-MPV → [SD] → HDAC → [remission] → relapse as BNS after 1.5 years→ HDAC → [SD] → BR → [remission] → relapse as BNS after 3 months → tirabrutinib	PR or CR	Herpes zoster (grade unclear)	12 months (no relapse)	(–)
Oyama et al. [8]	62M	R-CHOP, BR	Leptomeninges and cauda equina roots, bone marrow, axillary lymph node	Tirabrubinib	PR or CR	(–)	3 months (no relapse)	(–)
Saburi et al. [9]	66F	R-COP, R-CHOP	C2-4 cervical spinal cord, Th2-3 thoracic spinal cord	Tirabrubinib	CR	Pruritus (grade1)	10 months (no relapse)	(–)
Saburi et al. [10]	73M	R-CHOP, BR	Medulla, bilateral basal ganglia	R-MPV → [PD] → craniospinal irradiation (30.6 Gy) → progression to BNS after 2 years → tirabrutinib	CR	(–)	3 months (no relapse)	(–)
Hatasa et al. (current report)	75M	DRC, BD	Brachial plexus and nerve roots at C4-Th1 (cytology of CSF become negative before start of tirabrutinib)	Rituximab + steroid pulse, R-MPV → tirabrutinib	PR	Eczema (grade 1), decreased IgG level	25 months (no relapse)	Transient discontinuation due to COVID-19

Tirabrutinib demonstrated therapeutic effects in all cases, despite variations in BNS lesions. None of the patients experienced relapse during the follow-up period after the start of tirabrutinib treatment (median, 11 months; range, 3-25 months), suggesting persisting effects. Of the five reported patients, three had BNS refractory to chemotherapy prior to tirabrutinib treatment. One patient responded to bendamustine-rituximab but relapsed at three months, and subsequent tirabrutinib treatment exhibited a response lasting >12 months. These results suggested that tirabrutinib is effective even in patients who are resistant to other regimens, further suggesting that the duration of response may be longer than that for other therapies. Moreover, no tirabrutinib-associated AEs or only grade 1 AEs were recorded. Overall, these results suggest that tirabrutinib may be a promising treatment option for patients with BNS. Further comprehensive studies are needed to confirm the efficacy of tirabrutinib for the treatment of BNS.

Our case had some limitations in terms of diagnosis. We did not confirm the detailed etiology of PN because of the lack of anti-MAG antibodies or rarer autoantibodies. These tests are crucial for selecting the appropriate treatment for WM-related PN [1]. Therefore, they should be performed, regardless of whether they are covered by insurance. For the diagnosis of BNS, an MRI was performed only for the brain and cervical spine and not for the full spine. CSF tests based solely on cytological evaluation should be discouraged, and FCM and molecular tests should be evaluated. Because situations where BNS is suspected are rare, the diagnostic algorithm should be kept in mind in advance.

Finally, it is worth reconsidering the suitability of steroid pulse therapy and five courses of R-MPV for the current patient. Importantly, R-MPV contains vincristine, which is a neurotoxic agent, whereas corticosteroids have little or no effect on WM-associated PN [1]. We believe that it is reasonable to select a regimen with good CNS penetration for BNS; however, the main complaint in our patient was WM-associated PN. For BNS only, the goal of treatment is to clear the symptoms of the patient, while for asymptomatic BNS only observation is recommended [3]. Therefore, to provide symptom-oriented therapy rather than targeting the CSF findings, we should have considered changing the treatment in light of the "refractory" evaluation after two courses of R-MPV based on the lack of improvement in symptoms and MRI findings rather than the evaluation of "response" based on CSF findings.

## Conclusions

The possibility of concurrent BNS should be carefully investigated in patients with WM-associated PN. Repeated evaluations should be performed when lesions are close to the CNS or when the initial CSF findings are atypical, such as extremely high protein levels or slightly high leukocyte counts in patients with WM. Tirabrutinib may be the preferred treatment for WM-associated PN accompanied by BNS.
